# Nucleus Pulposus Cell Conditioned Medium Promotes Mesenchymal Stem Cell Differentiation into Nucleus Pulposus-Like Cells under Hypoxic Conditions

**DOI:** 10.1155/2020/8882549

**Published:** 2020-12-23

**Authors:** Arjun Sinkemani, Feng Wang, Zhiyang Xie, Lu Chen, Cong Zhang, Xiaotao Wu

**Affiliations:** Department of Spine Surgery, Zhongda Hospital, Southeast University School of Medicine, 87# Dingjiaqiao Road, 210009 Nanjing, China

## Abstract

Low back pain (LBP) is a major physical and socioeconomic challenge worldwide. Nucleus pulposus (NP) is directly associated with LBP due to intervertebral disc (IVD) degeneration. IVD degeneration is mainly caused by structural and matrix-related changes within the IVD occurring during aging and degeneration. Mesenchymal stem cells (MSCs) can differentiate into multiple mesenchymal lineages under specific stimulatory conditions. This study is aimed at evaluating the effectiveness of the nucleus pulposus cell (NPC) conditioned medium for promoting the expression of MSCs and at confirming the expression of healthy NP phenotypic markers recently recommended by the Spine Research Interest Group. Expression was investigated using quantitative polymerase chain reaction (qPCR) and western blotting under normoxic and hypoxic conditions. qPCR and western blotting demonstrated significant upregulation of NP marker expression in MSCs cultured under hypoxic conditions and treated with the 50% or 100% NPC conditioned medium, compared with those cultured under normoxic conditions. Upregulation was highest in the presence of the 100% NPC conditioned medium compared with the control group (aggrecan, *p* < 0.01; brachyury, *p* < 0.05; collagen II, *p* < 0.001; KRT8, *p* < 0.01; KRT19, *p* < 0.001; and Shh, *p* < 0.01). The expression levels of genes in MSCs treated with the 50% NPC conditioned medium also showed upregulation compared with the control group (collagen II, *p* < 0.05; KRT8, *p* < 0.05; and KRT19, *p* < 0.01). These findings suggested that the NPC conditioned medium stimulated MSC differentiation into an NP-like phenotype with distinct characteristics. The results could inform strategies for IVD regeneration.

## 1. Introduction

Low back pain (LBP) is a major cause of disability worldwide [1], which affects more than 84% of people at least once during their lifetime [2]. Although it is presumably multifactorial, the underlying etiology of LBP is unknown. Intervertebral disc (IVD) degeneration is the main clinical presentation. The primary cause of disc degeneration is tissue breakdown, primarily due to senescence, poor diet, genetic inheritance, or injury [3]. IVD degeneration is a process and is often caused by biomechanical changes within the IVD. The causes of IVD degeneration have not been fully elucidated but are presumed to include environmental and genetic factors [4]. During disc degeneration, various homeostatic alterations occur in resident cells, which result in degradative tissue anabolism and elevated tissue catabolism [5]. Disc degeneration can be caused by disc tissue dehydration, proteoglycan loss, fibrotic tissue formation, and disc height reduction [3, 6]. The gelatinous extracellular matrix breaks down, leading to fibrotic tissue formation that strains nucleus pulposus cells (NPCs) [7, 8].

Current treatment strategies provide symptomatic pain relief rather than the resolution of the underlying etiology of IVD degeneration. Spinal fusion and discectomy are the most widely used surgical treatments for LBP. In these surgical procedures, degenerated or herniated disc is removed, and nearby discs are fused with metal rods and bone cement; the latter is used in vertebroplasty and balloon kyphoplasty [9, 10]. Although the procedures are designed to relieve pain, patients eventually experience persistent pain and reduced spinal mobility [11]. These procedures may accelerate degeneration in some IVD segments due to biomechanical changes [12, 13]. Because of the poor long-term efficacy of current treatments and increasing prevalence of IVD degeneration, there is a need to address the underlying pathogenesis of IVD degeneration and restore normal IVD function, rather than simply achieve pain reduction. Accordingly, biological and cell-based therapies are necessary as alternative treatments for IVD degeneration.

Cell-based therapies include the implantation of autologous NPCs or stem cells in the degenerated discs, with the aim of restoring IVD metabolic homeostasis and reducing inflammation by replacing NPCs in the early stage of IVD degeneration. Mesenchymal stem cells (MSCs) are an important option for cell-based therapy. MSCs are the most frequently studied and widely used cells for regeneration purposes [14]. Studies have shown that MSCs can be differentiated into NP-like cells and may provide a good cell source for IVD regeneration. Furthermore, some publications have demonstrated that MSCs cocultured with the NPC conditioned medium can differentiate into NP-like cells and acquire a phenotype similar to that of NPCs [15]. Moreover, MSCs cultured in the notochordal cell conditioned medium synthesize high levels of proteoglycans within the IVD, presumably through stimulation of NPCs by metabolites, growth factors, and extracellular matrix proteins [16–18]. Therefore, MSCs have a high capacity for IVD regeneration [19].

The Spine Research Interest Group of the Orthopedic Research Society stated that stable expression of HIF-1*α*, Glut-1, aggrecan/collagen II (ratio > 20), Shh, brachyury, KRT18/19, CA12, and CD24 is necessary for spinal health [20]. However, there is a need for further investigation at the gene and protein levels to better understand MSC differentiation into NP-like cells. Therefore, the aim of this study was to determine the effects of the NPC conditioned medium on the expression of MSCs cultured under normoxic and hypoxic conditions. Both the mRNA and protein levels were assessed using quantitative polymerase chain reaction (qPCR) and western blotting assays, to explore changes in gene and protein expression that might promote MSC differentiation into NP-like cells, and thus aid treatment of IVD degeneration.

## 2. Materials and Methods

### 2.1. Isolation and Culture of NPCs

The Institutional Animal Care and Use Committee of Southeast University (Nanjing, China) approved all animal experiments performed in this study. Twenty male Sprague–Dawley rats (8 weeks old, 200–300 g) were euthanized with CO_2_. The NP tissues were isolated as described in our previous study [21]. NPCs were cultured in Dulbecco's modified Eagle's medium (DMEM)/F-12 containing 10% fetal bovine serum (FBS; Gibco, Grand Island, NY, USA) and 1% penicillin-streptomycin solution under 5% CO_2_ and 21% O_2_ at 37°C. Cells were harvested using 0.25% trypsin-EDTA (1 mM) solution (Life Technologies, Carlsbad, CA, USA) and subcultured (1 : 2); cells were collected at passages 1–3. The culture medium was changed at 3-day intervals. The NPC conditioned medium (passage 3) was used in the experiments.

### 2.2. MSC Culture in the 50% or 100% NPC Conditioned Medium

Sprague–Dawley rat bone marrow-derived MSCs were purchased from Cyagen Biosciences (Suzhou, China). The MSCs were cultured in DMEM/F-12 containing 10% FBS (Gibco) and 1% penicillin-streptomycin solution. All cells were cultured under 5% CO_2_ and 21% O_2_ at 37°C. The culture medium was changed at 3-day intervals. Cells from passage 5 were used in our studies. MSCs were cultured for 7 days with the 50% or 100% NPC conditioned medium in the experimental groups, while the control group MSCs were cultured in DMEM/F-12 containing 10% FBS (Gibco) and 1% penicillin-streptomycin solution. The culture medium for the control group and conditioned medium for the experimental groups were changed twice per week.

### 2.3. Cell Culture under Hypoxic Conditions

NPCs were cultured in a hypoxic environment (3131 Incubator; Thermo Fisher Scientific, Waltham, MA, USA) under conditions of 93% N_2_, 5% CO_2_, and 2% O_2_ at 37°C [22]. The NPCs were cultured in fresh DMEM/F-12, supplemented with 10% FBS and 1% penicillin-streptomycin solution, and then subjected to hypoxic conditions for 3 days. As noted above, the NPC conditioned medium (passage 3) was used in the experiments. MSCs were also cultured in a hypoxic environment (3131 Incubator; Thermo Fisher Scientific) under conditions of 93% N_2_, 5% CO_2_, and 2% O_2_ at 37°C [22]. MSCs were cultured for 7 days with the 50% or 100% NPC conditioned medium in the experimental groups, while the control group MSCs were cultured in DMEM/F-12 containing 10% FBS (Gibco) and 1% penicillin-streptomycin solution. The culture medium for the control group and conditioned medium for the experimental groups were changed twice per week.

### 2.4. Quantitative Polymerase Chain Reaction

Total mRNA was extracted from NPCs and MSCs, both of which had been cultured under normoxic and hypoxic conditions in 6 cm plates, at days 3 and 7, respectively. mRNA extraction was performed using a Universal RNA Extraction Kit (9767; Takara, Dalian, China). Reverse transcription was performed using the PrimeScript RT Reagent Kit with gDNA Eraser (RR047; Takara) in accordance with the manufacturer's instructions. The qPCR analysis was carried out using SYBR Green PCR Master Mix (Roche Applied Science, Penzberg, Germany) in the StepOnePlus PCR System (Applied Biosystems, Foster City, CA, USA). The qPCR assay mixture contained 0.5 *μ*L of reverse primer (10 *μ*M), 2 *μ*L of cDNA template, 0.5 *μ*L of forward primer (10 *μ*M), 10 *μ*L of SYBR Green solution, and 7 *μ*L of dH_2_O. The gene-specific primer sequences are listed in Table 1. The target gene expression levels were normalized to *β*-actin using the 2^-*ΔΔ*Ct^ cycle threshold method.

### 2.5. Protein Extraction and Western Blotting

NPCs and MSCs cultured under normoxic and hypoxic conditions were placed on ice, harvested, and washed with cold phosphate-buffered saline (PBS) solution at days 3 and 7, respectively. Total protein was extracted using the whole-cell lysis assay buffer (KeyGen, Nanjing, China). Protein concentrations were determined using the bicinchoninic acid assay (Beyotime, Jiangsu, China). Proteins were separated via 10% SDS-PAGE and transferred onto polyvinylidene difluoride membranes (EMD Millipore, Burlington, MA, USA). The membranes were blocked with 5% bovine serum albumin (BSA) for 1.5 h and then incubated overnight with primary antibodies (Table 2) at 4°C with gentle shaking. Subsequently, membranes were incubated with a secondary antibody (ab6721, 1 : 5000; Abcam, Cambridge, UK) which was diluted in 5% BSA for 1 h at room temperature. Immunolabeling was detected using the SuperSignal West FEMTO Chemiluminescent Substrate (Thermo Fisher Scientific). Relative protein expression levels were determined by quantitative densitometric analysis and normalized to the level of *β*-actin.

### 2.6. Immunofluorescence Staining Microscopy

NPCs were plated in 12-well plates and maintained under hypoxic and normoxic conditions for 3 days. The cells were then fixed with 4% paraformaldehyde for 20 min. NPCs on glass-bottom slides (Cellvis, Mountain View, CA, USA) were blocked in a buffer containing 5% BSA and 0.1% Triton X-100 in PBS for 20 min. Then, NPCs were washed three times and incubated at 4°C overnight with primary antibodies (Table 2). After the cells had been washed, they were incubated with a secondary antibody (Alexa Fluor 594 goat anti-rabbit, 1 : 1000; Invitrogen, Carlsbad, CA, USA) for 1 h at room temperature. Cell nuclei were counterstained with 4,6-diamidino-2-phenylindole (DAPI; Sigma-Aldrich, St. Louis, MO, USA) for 15 min at room temperature and observed using a fluorescence microscope (Olympus, Tokyo, Japan).

### 2.7. Statistical Analysis

Results are expressed as means ± standard deviations from three independent experiments. Statistical analyses were performed using GraphPad Prism software (ver. 7.04; GraphPad Software, Inc., La Jolla, CA, USA), and differences among groups were analyzed using unpaired Student's *t*-tests. Differences with *p* < 0.05 were considered statistically significant.

## 3. Results

### 3.1. Gene Expression in NPCs Cultured under Normoxic and Hypoxic Conditions

**Figure 1:**
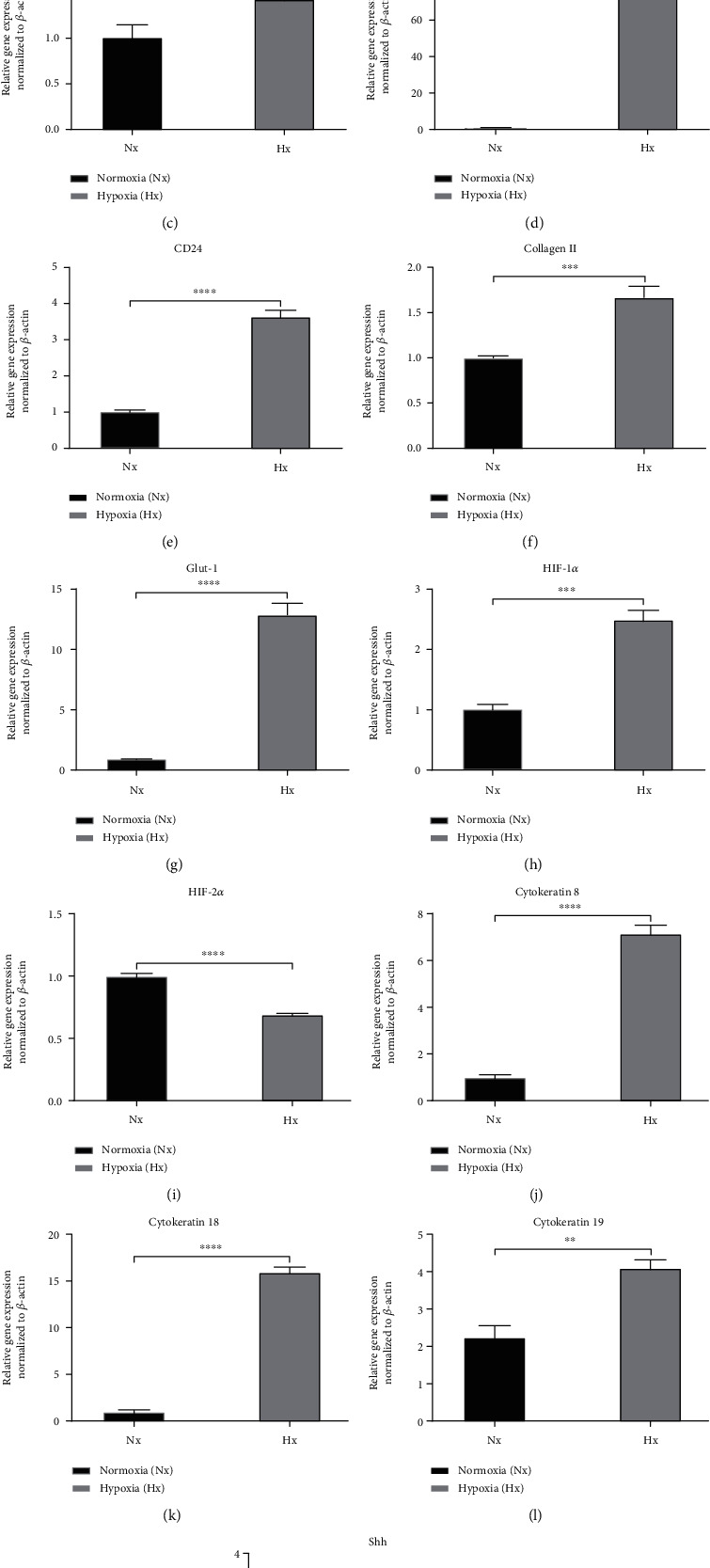
Markers recommended for defining the young healthy NP phenotype. Relative gene expression by qPCR in NPCs cultured under normoxic and hypoxic conditions. Gene expression levels for each sample were normalized against the housekeeping gene *β*-actin. (a) Aggrecan, (b) brachyury, (c) carbonic anhydrase 3, (d) carbonic anhydrase 12, (e) CD24, (f) collagen II, (g) Glut-1, (h) HIF-1*α*, (i) HIF-2*α*, (j) KRT8, (k) KRT18, (l) KRT19, and (m) Shh. ^∗^*p* < 0.05, ^∗∗^*p* < 0.01, ^∗∗∗^*p* < 0.001, and ^∗∗∗∗^*p* < 0.0001.

qPCR analysis was performed to characterize gene expression in Sprague–Dawley rat NPCs cultured under normoxic and hypoxic conditions. The results showed that the recommended young healthy NPC phenotype markers were expressed in both the normoxic and hypoxic conditions. The gene expression of NPCs was generally significantly higher in hypoxic conditions than in normoxic conditions, although HIF-2*α* exhibited higher expression in normoxic conditions (*p* < 0.0001). The expression level of the CA3 gene was lower than that of KRT19, Shh, aggrecan, brachyury, collagen II, and HIF-1*α* in hypoxic conditions (*p* < 0.01) (Figure 1). In contrast, CA12, CD24, Glut-1, KRT8, and KRT18 showed significantly higher expression in hypoxic conditions (*p* < 0.0001) (Figure 1).

### 3.2. Protein Expression in NPCs Cultured under Normoxic and Hypoxic Conditions

**Figure 2:**
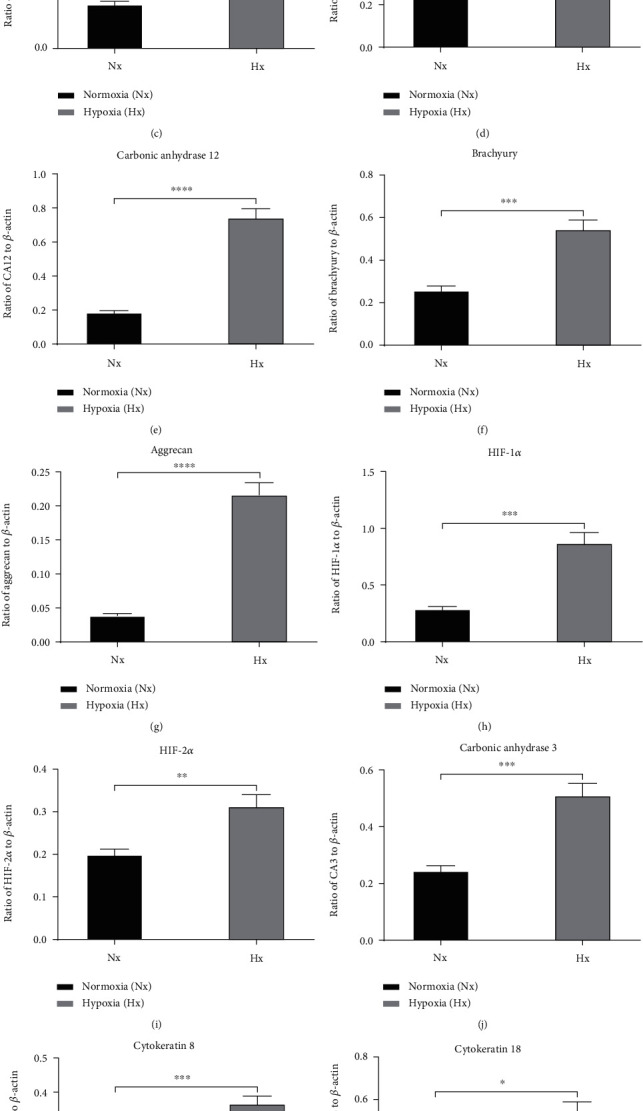
(a) Relative protein expression by western blotting in NPCs cultured under normoxic and hypoxic conditions. Protein expression levels for each sample were normalized against the housekeeping gene *β*-actin. (b) Collagen II, (c) Glut-1, (d) CD24, (e) carbonic anhydrase 12, (f) brachyury, (g) aggrecan, (h) HIF-1*α*, (i) HIF-2*α*, (j) carbonic anhydrase 3, (k) KRT8, (l) KRT18, (m) KRT19, and (n) Shh. ^∗^*p* < 0.05, ^∗∗^*p* < 0.01, ^∗∗∗^*p* < 0.001, and ^∗∗∗∗^*p* < 0.0001.

Western blotting analysis was performed to investigate whether the proteins were also expressed in Sprague–Dawley rat NPCs in both the normoxic and hypoxic conditions. Notably, the recommended young healthy NPC phenotype markers were also expressed in both the normoxic and hypoxic conditions. The results of western blotting analysis were consistent with the results of qPCR, in that expression levels were higher in hypoxic conditions than normoxic conditions. The expression levels of KRT18, KRT19, and Shh were lower in hypoxic conditions (*p* < 0.05), whereas collagen II, CD24, HIF-2*α*, Glut-1, brachyury, HIF-1*α*, CA3, KRT8, aggrecan, and CA12 were not (Figure 2). Furthermore, CA12 and aggrecan expression levels showed significant upregulation (*p* < 0.0001) in hypoxic conditions (Figure 2).

### 3.3. Immunofluorescence Staining Showing Protein Expression Patterns in NPCs

**Figure 3:**
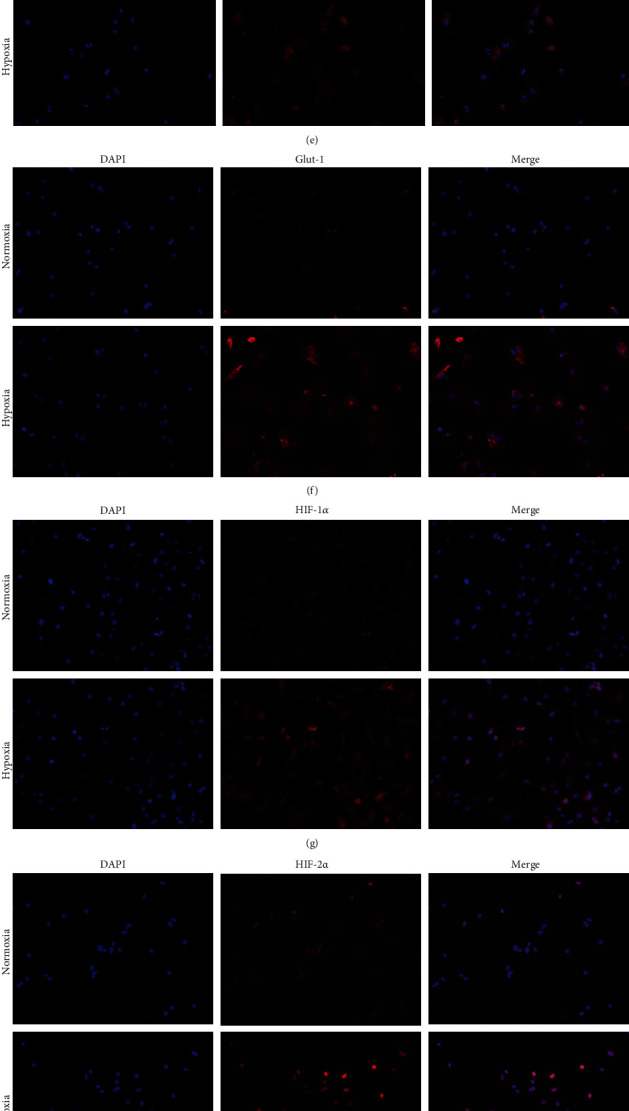
Relative protein expression in NPCs cultured under normoxic and hypoxic conditions, measured by immunofluorescence. (a) Aggrecan, (b) brachyury, (c) carbonic anhydrase 3, (d) CD24, (e) collagen II, (f) Glut-1, (g) HIF-1*α*, (h) HIF-2*α*, (i) KRT8, (j) KRT18, (k) KRT19, and (l) Shh.

Immunofluorescence analysis was performed to identify protein expression in NPCs. All recommended young healthy NP phenotype markers were expressed in NPCs cultured under normoxic and hypoxic conditions. Immunofluorescence staining showed significantly increased expression levels in hypoxic conditions. Aggrecan, brachyury, carbonic anhydrase 3, CD24, collagen II, Glut-1, HIF-1*α*, HIF-2*α*, KRT8, KRT18, KRT19, and Shh showed extensive staining under hypoxic conditions (Figure 3).

### 3.4. Gene Expression in MSCs Cultured under Normoxic and Hypoxic Conditions

**Figure 4:**
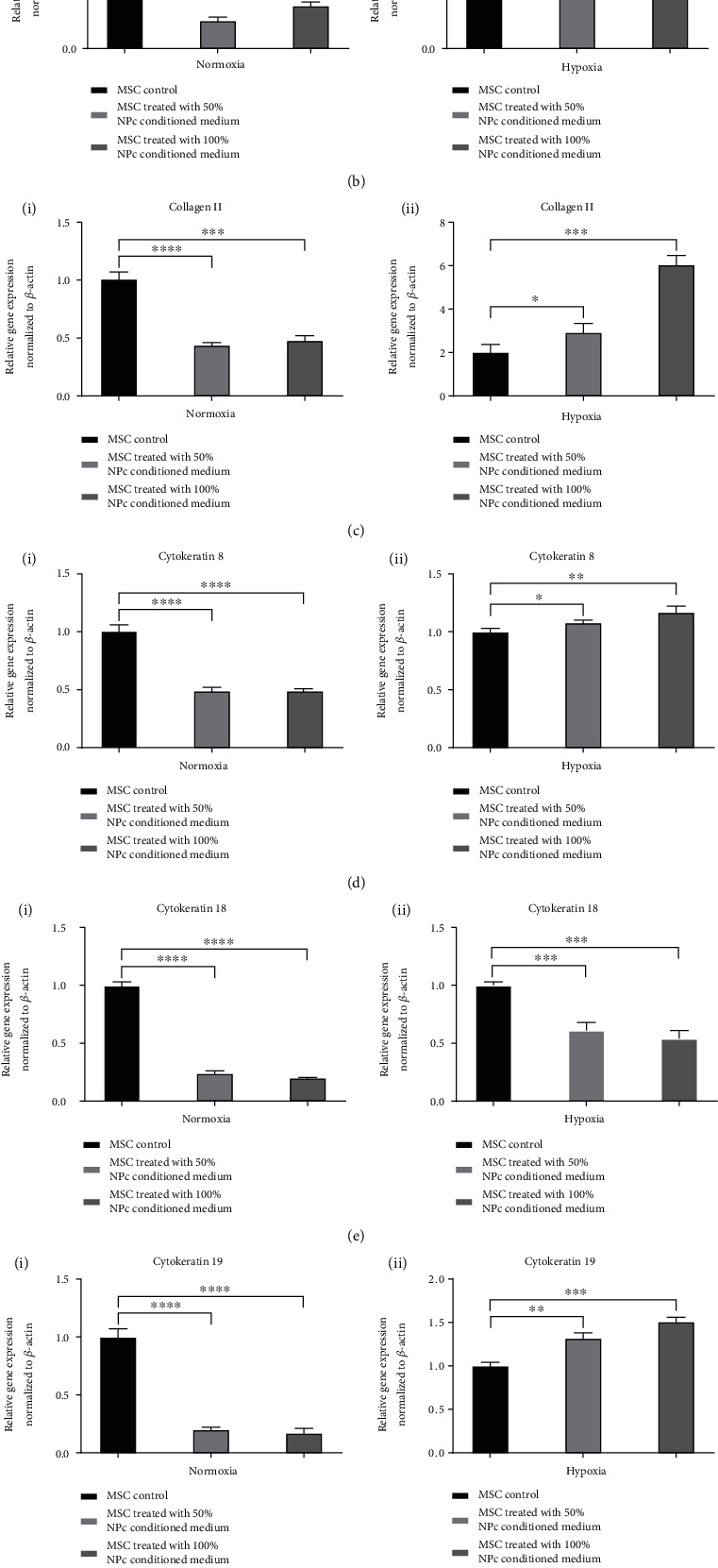
qPCR assessment of relative gene expression in MSCs under normoxic and hypoxic conditions with the 50% or 100% NPC conditioned medium. Gene expression levels for each sample were normalized against the housekeeping gene *β*-actin. (a) Aggrecan: (i) normoxia, (ii) hypoxia; (b) brachyury: (i) normoxia, (ii) hypoxia; (c) collagen II: (i) normoxia, (ii) hypoxia; (d) KRT8: (i) normoxia, (ii) hypoxia; (e) KRT18: (i) normoxia, (ii) hypoxia; (f) KRT19: (i) normoxia, (ii) hypoxia; and (g) Shh: (i) normoxia, (ii) hypoxia. ^∗^*p* < 0.05, ^∗∗^*p* < 0.01, ^∗∗∗^*p* < 0.001, and ^∗∗∗∗^*p* < 0.0001.

qPCR analysis was performed to characterize gene expression in Sprague–Dawley rat bone marrow-derived MSCs cultured under normoxic and hypoxic conditions. The gene expression of MSCs was upregulated in hypoxic conditions with the 50% or 100% NPC conditioned medium, compared with normoxic conditions. The upregulation was greatest with the 100% NPC conditioned medium compared with the control group (aggrecan, *p* < 0.01; brachyury, *p* < 0.05; collagen II, *p* < 0.001; KRT8, *p* < 0.01; KRT19, *p* < 0.001; and Shh, *p* < 0.01). The expression levels in the 50% NPC conditioned medium condition were also upregulated compared with the control group (collagen II, *p* < 0.05; KRT8, *p* < 0.05; and KRT19, *p* < 0.01). However, the expression level of KRT18 was opposite to that of the other respective genes, in that it was lower under both the normoxic and hypoxic conditions with the 50% or 100% NPC conditioned medium compared with the control group (Figure 4).

### 3.5. Protein Expression in MSCs Cultured under Normoxic and Hypoxic Conditions

**Figure 5:**
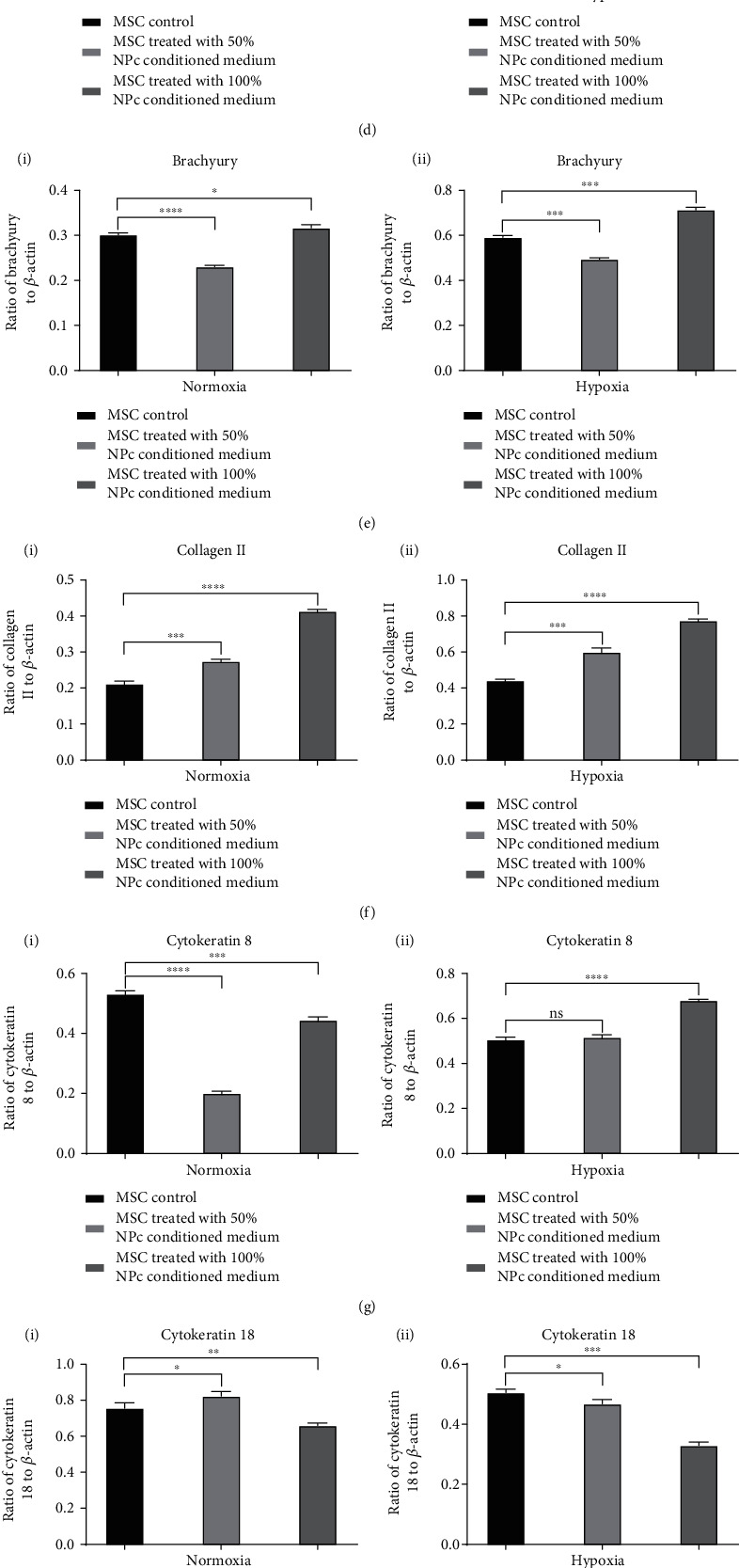
(a–c) Western blotting assessment of relative protein expression in MSCs under normoxic and hypoxic conditions with the 50% or 100% NPC conditioned medium. Protein expression levels for each sample were normalized against the housekeeping gene *β*-actin. (d) Aggrecan: (i) normoxia, (ii) hypoxia; (e) brachyury: (i) normoxia, (ii) hypoxia; (f) collagen II: (i) normoxia, (ii) hypoxia; (g) KRT8: (i) normoxia, (ii) hypoxia; (h) KRT18: (i) normoxia, (ii) hypoxia; (i) KRT19: (i) normoxia, (ii) hypoxia; and (j) Shh: (i) normoxia, (ii) hypoxia. ^∗^*p* < 0.05, ^∗∗^*p* < 0.01, ^∗∗∗^*p* < 0.001, and ^∗∗∗∗^*p* < 0.0001.

Western blotting analysis was performed to investigate whether the proteins were also expressed in rat bone marrow-derived MSCs in both the normoxic and hypoxic conditions. Importantly, the expression levels were upregulated upon treatment with the 50% or 100% NPC conditioned medium. The results of western blotting analysis were consistent with those of qPCR, in that expression levels were higher in hypoxic conditions than normoxic conditions. The upregulation was greatest with the 100% NPC conditioned medium compared with the control group (aggrecan, *p* < 0.0001; brachyury, *p* < 0.001; collagen II, *p* < 0.0001; KRT8, *p* < 0.0001; KRT19, *p* < 0.0001; and Shh, *p* < 0.01). The expression levels in the 50% NPC conditioned medium condition were also upregulated compared with the control group (aggrecan, *p* < 0.01; collagen II, *p* < 0.001; KRT19, *p* < 0.0001; and Shh, *p* < 0.05). Moreover, the expression level of KRT18 was consistent with the results of qPCR analysis, in that it was lower under both the normoxic and hypoxic conditions with the 50% or 100% NPC conditioned medium compared with the control group (Figure 5).

## 4. Discussion

Many studies have been performed on IVD, but the origins of NP and NPCs remain unclear. However, normal NP can be characterized by molecular profiling. There is evidence that mature NPCs differentiated through notochordal lineages and that all cells (e.g., chondrocyte-like cells) in adults are also differentiated from notochordal lineages with variable morphology and size during maturation. These studies also suggested that certain markers are more specific to NPCs [23–30]. NPs are located in hypoxic environments and are adapted for the IVD microenvironment.

MSCs are a potential cell type for IVD regeneration. Among the available cell sources, bone marrow-derived MSCs are frequently used. Many studies have reported that MSCs exhibit a robust differentiation ability toward the NPC phenotype, which has proven useful in clinical trials. To obtain a better understanding of MSC differentiation into NP-like cells, we investigated the effects of the 50% or 100% NPC conditioned medium on MSCs. Some previous studies showed that the notochordal cells and notochordal cell conditioned medium had positive effects in the treatment of IVD degeneration [31, 32]. However, it remains unclear whether these positive effects apply to MSCs. Aggrecan and collagen II show enhanced expression associated with a healthy NPC phenotype, whereas MSCs showed stimulatory effects (e.g., upregulated deposition of proteoglycan in IVD) when exposed to the notochordal cell conditioned medium [33].

Our study was conducted under both the normoxic and hypoxic conditions. Hypoxic conditions have shown an ability to support MSC differentiation toward an NPC phenotype [34]. We have cultured NPCs and MSCs under both the normoxic and hypoxic conditions. We used the 50% or 100% NPC conditioned medium (passage 3) for the treatment of MSCs. Treatment with the NPC conditioned medium had a more positive impact on MSC differentiation. Aggrecan and collagen II are distinctly expressed in the normal NP, and the loss of aggrecan synthesis has been observed in IVD degeneration [35]. There is also evidence that normoxic-independent stabilization of HIF-1*α* drives metabolic glycolysis, which can aid in regulating aggrecan gene expression in NPCs [36]. During IVD degeneration, increased cell senescence has been reported, along with reduced production of aggrecan and collagen II [37]. In our study, aggrecan and collagen II showed distinct upregulation under hypoxic conditions, following treatment with the 50% or 100% NPC conditioned medium. Collectively, our study showed that the NPC conditioned medium may promote regeneration through MSC differentiation toward healthy NPC phenotypes.

The results of our study are consistent with those of some other studies concerning the regenerative effects of notochordal cells; specifically, the NPC conditioned medium was presumed to contain certain nutritional constituents and secretory growth factors [16, 38]. Our previous study demonstrated that MSCs play a vital role in preserving normal NP tissue stability by maintaining physiological processes [39]. However, that study also suggested that NPC senescence leads to IVD degeneration, where preventing this senescence can reduce IVD degeneration [40]. Notably, our study suggested that NPC senescence can be inhibited by MSC treatment with the 100% NPC conditioned medium under hypoxic conditions.

To identify the recommended phenotype markers of young healthy NPCs, we also examined the expression of NPC markers under normoxic and hypoxic culture conditions. Our study showed significant upregulation of NP markers when NPCs were cultured in hypoxic conditions. FOX1, PAX1, and CA12 are highly expressed in NPCs [30], while HIF-1*α* in NPCs is metabolically adapted to conditions involving low oxygen and nutrition deficiency [36]. The NP is the largest avascular tissue in the IVD, which is physiologically adapted to survive in a hypoxic microenvironment through the expression of HIF-1*α* and HIF-2*α*. The expression of HIF-2*α* is regulated by the NF-*κ*B pathway, where HIF-2*α* levels are reduced under hypoxic conditions [41, 42]. Our results suggested that MSC explantation can enhance differentiation toward NPC phenotypes for IVD regeneration. The reduction of the extracellular matrix is another important consideration. The balance between extracellular matrix anabolism and catabolism mediated by disc cells is influenced by proinflammatory cytokine instability during IVD degeneration [43, 44]. Therefore, inhibiting the pathological process involving these inflammatory cytokines might stimulate MSC differentiation, resulting in enhanced extracellular matrix deposition. Notably, our results showed upregulated expression of aggrecan and collagen II, which can promote matrix formation.

Shh and brachyury are expressed in the developing notochord, where they are required for notochordal sheath formation and NP patterning [45–47]. Transcriptional factors expressed by the notochord, as well as the presence of NP patterning factors (Shh and brachyury), guide NPC differentiation and survival. The expression levels of Shh and brachyury are informative regarding NPC ontology [47]. Shh and brachyury levels are downregulated with aging and IVD degeneration [30]. Increased expression levels of brachyury, aggrecan, and chondroitin sulfate are caused by Wnt signaling activation in aged discs [48]. Some studies have reported that brachyury is also expressed in some mature NPs [49, 50]. In a study by Tang et al., brachyury was expressed in developing human NP tissue [51]. Therefore, expression of the NP markers Shh and brachyury suggested the presence of notochordal cell markers in our study, confirming the findings of previous studies [29, 30, 52]. In addition, MSCs treated with the 100% NPC conditioned medium showed considerably higher expression levels of the brachyury and Shh genes. These results were presumed to be indicative of MSC differentiation toward an NP-like phenotype.

Cytokeratins (KRT8, KRT18, and KRT19) were previously identified as markers for rat NPCs. These genes are also expressed in normal epithelial cells and are presumed to be human notochord-specific markers, although KRT19 alone was confirmed in human NPCs. However, KRT19 exhibited reduced expression in aging discs [24, 53, 54]. A microarray study by Minogue et al. identified NP-specific expression patterns of several genes, including KRT8, KRT18, and KRT19, in bovine NPCs [49]. Cytokeratins have unique cytoskeletal functions that aid maintenance of stable cell structures, regulate Fas-mediated apoptosis, and modulate both cell size and protein synthesis [55]. They also play important roles in tissue development and differentiation. KRT8, KRT18, and KRT19 were identified in both the rat and human NPCs [24, 25, 56]. Rodrigues-Pinto et al. reported that KRT8, KRT18, and KRT19 were expressed in IVD notochordal cells in all developmental stages [57]. In a recent study, KRT8 and KRT18 were detected in NPCs regardless of age or degeneration, whereas KRT19 expression was enhanced in aged IVD [29]. Indeed, KRT8, KRT18, and KRT19 showed significantly higher expression in hypoxic conditions in NPCs. Those results were consistent with MSC expression, with the exception of KRT18. qPCR and western blotting analyses of KRT18 showed lower expression under both the normoxic and hypoxic conditions. The hypoxic environment of the IVD promotes ontogeny and maintenance of cell function.

In this study, we evaluated recommended NP phenotypic markers and investigated the effects of the NPC conditioned medium on MSCs in both the normoxic and hypoxic environments. We concluded that these recommended phenotypic markers were expressed in both the normoxic and hypoxic culture conditions and that the NPC conditioned medium contains a secretory factor that influences gene and protein expression in MSCs. Thus, the secretory factor presumably promoted MSC differentiation into an NP-like phenotype.

## 5. Conclusion

Our study provided data that could aid the identification of the healthy NPC phenotype. We concluded that NPCs express functionally active HIF-1*α*, HIF-2*α*, Glut-1, aggrecan, collagen II, Shh, brachyury, KRT8, KRT18, KRT19, CA3, CA12, and CD24 under both the normoxic and hypoxic conditions. Furthermore, the NPC conditioned medium promoted MSC differentiation into NP-like cells. Finally, this study showed that NPCs and MSCs can adapt to hypoxic microenvironments, in which hypoxia stimulates these cells to maintain their specific characteristics. Our study provides a scientific basis for cell-based therapy in the treatment of IVD degeneration.

## Figures and Tables

**Table 1:** Primer sequences for gene expression analysis used in qPCR.

Genes	Forward (5′-3′)	Reverse (5′-3′)
HIF-1*α*	GCAACTGCCACCACTGATGA	GCTGTCCGACTGTGAGTACC
HIF-2*α*	GCACCAGCAGTTCACACTTG	CTGACGGTCTTGTCAGGCAT
Glut-1	GCTGTGGCTGGCTTCTCTAA	CCGGAAGCGATCTCATCGAA
Shh	TATGAGGGTCGAGCAGTGGA	AGTGGATGCGAGCTTTGGAT
Brachyury	TCTAGGCACAACATGGCAGG	AAATTGGACCACAGCCTCGT
Aggrecan	TGGCCTGCCTGACTTTAGTG	CCTGAACCACTGACGCTGAT
Collagen II	GGCCAGGATGCCCGAAAATTA	ACCCCTCTCTCCCTTGTCAC
Carbonic anhydrase 3	TGGTTCACTGGAACCCGAAG	GAGCCTCCTTGCCCTTAGTC
Carbonic anhydrase 12	TCCGACAAGGACTGCTTACC	GGAGATCCCAAGGACACCAG
CD24	AGTAATTGACACGGGCCAGG	TGGGAAATGCTTTGCCGTTG
Cytokeratin 8	CCCGGCTTCAGCTATGGAAT	AGCCTTGGTGCGGCTATAAG
Cytokeratin 18	GACCCAGGAATACGAAGCCC	ACTTTGCCATCCACGACCTT
Cytokeratin 19	GAGATCGCCACCTACCGAAG	GGAAGGGCTGGTGTGAACTT

qPCR: quantitative polymerase chain reaction.

**Table 2:** Antibodies used in WB and IF.

Target	Antibody (company, order number)	WB usage	IF usage
HIF-1*α*	Abcam, ab2185	1 : 1000	1 : 1000
HIF-2*α*	Abcam, ab199	1 : 1000	1 : 1000
Glut-1	Abcam, ab15309	1 : 250	1 : 250
Shh	Abcam, ab19897	1 : 250	1 : 50
Brachyury	Bioss, bs-10669R	1 : 250	1 : 250
Aggrecan	Bioss, bs-11655R	1 : 250	1 : 250
Collagen II	Abcam, ab116242	1 : 1000	1 : 1000
Carbonic anhydrase 3	Abcam, ab196835	1 : 1000	1 : 100
Carbonic anhydrase 12	(D78E4) CST, 5865	1 : 1000	
Carbonic anhydrase 12	Bioss, bs-6025R		1 : 250
CD24	Proteintech, 10600-1-AP	1 : 1000	
CD24	Bioss, bs-4891R		1 : 250
Cytokeratin 8	Abcam, ab59400	1 : 1000	1 : 500
Cytokeratin 18	Abcam, ab181597	1 : 1000	1 : 100
Cytokeratin 19	Abcam, ab84632	1 : 500	1 : 500
*β*-Actin	Abcam, ab8227	1 : 2000	

WB: western blotting; IF: immunofluorescence.

## Data Availability

Data are available on request (detail contact information: Arjun Sinkemani, sinkemani@hotmail.com).

## References

[1] Stewart W. F., Ricci J. A., Chee E., Morganstein D., Lipton R. (2003). Lost productive time and cost due to common pain conditions in the US workforce. *JAMA*.

[2] Walker B. F. (2000). The prevalence of low back pain: a systematic review of the literature from 1966 to 1998. *Journal of Spinal Disorders*.

[3] Adams M. A., Roughley P. J. (2006). What is intervertebral disc degeneration, and what causes it?. *Spine (Phila Pa 1976)*.

[4] Mayer J. E., Iatridis J. C., Chan D., Qureshi S. A., Gottesman O., Hecht A. C. (2013). Genetic polymorphisms associated with intervertebral disc degeneration. *The Spine Journal*.

[5] Le Maitre C. L., Pockert A., Buttle D. J., Freemont A. J., Hoyland J. A. (2007). Matrix synthesis and degradation in human intervertebral disc degeneration. *Biochemical Society Transactions*.

[6] Urban J. P., Roberts S. (2003). Degeneration of the intervertebral disc. *Arthritis Research & Therapy*.

[7] Priyadarshani P., Li Y., Yao L. (2016). Advances in biological therapy for nucleus pulposus regeneration. *Osteoarthritis and Cartilage*.

[8] Phillips K. L., Chiverton N., Michael A. L. (2013). The cytokine and chemokine expression profile of nucleus pulposus cells: implications for degeneration and regeneration of the intervertebral disc. *Arthritis Research & Therapy*.

[9] Zhang Y., An H. S., Tannoury C., Thonar E. J., Freedman M. K., Anderson D. G. (2008). Biological treatment for degenerative disc disease: implications for the field of physical medicine and rehabilitation. *American Journal of Physical Medicine & Rehabilitation*.

[10] Lewis G. (2011). Viscoelastic properties of injectable bone cements for orthopaedic applications: state-of-the-art review. *Journal of Biomedical Materials Research. Part B, Applied Biomaterials*.

[11] Ghiselli G., Wang J. C., Bhatia N. N., Hsu W. K., Dawson E. G. (2004). Adjacent segment degeneration in the lumbar spine. *Journal Bone Joint Surgery*.

[12] Gillet P. (2003). The fate of the adjacent motion segments after lumbar fusion. *Journal of Spinal Disorders & Techniques*.

[13] Berg S., Tropp H. T., Leivseth G. (2011). Disc height and motion patterns in the lumbar spine in patients operated with total disc replacement or fusion for discogenic back pain. Results from a randomized controlled trial. *The Spine Journal*.

[14] Clarke L. E., Richardson S. M., Hoyland J. A. (2015). Harnessing the potential of mesenchymal stem cells for IVD regeneration. *Current Stem Cell Research & Therapy*.

[15] Arkesteijn I. T., Smolders L. A., Spillekom S. (2015). Effect of coculturing canine notochordal, nucleus pulposus and mesenchymal stromal cells for intervertebral disc regeneration. *Arthritis Research & Therapy*.

[16] Erwin W. M., Islam D., Inman R. D., Fehlings M. G., Tsui F. W. (2011). Notochordal cells protect nucleus pulposus cells from degradation and apoptosis: implications for the mechanisms of intervertebral disc degeneration. *Arthritis Research & Therapy*.

[17] Korecki C. L., Taboas J. M., Tuan R. S., Iatridis J. C. (2010). Notochordal cell conditioned medium stimulates mesenchymal stem cell differentiation toward a young nucleus pulposus phenotype. *Stem Cell Research & Therapy*.

[18] Purmessur D., Schek R. M., Abbott R. D., Ballif B. A., Godburn K. E., Iatridis J. C. (2011). Notochordal conditioned media from tissue increases proteoglycan accumulation and promotes a healthy nucleus pulposus phenotype in human mesenchymal stem cells. *Arthritis Research & Therapy*.

[19] Navaro Y., Bleich-Kimelman N., Hazanov L. (2015). Matrix stiffness determines the fate of nucleus pulposus-derived stem cells. *Biomaterials*.

[20] Risbud M. V., Schoepflin Z. R., Mwale F. (2015). Defining the phenotype of young healthy nucleus pulposus cells: recommendations of the Spine Research Interest Group at the 2014 annual ORS meeting. *Journal of Orthopaedic Research*.

[21] Wang F., Cai F., Shi R., Wei J. N., Wu X. T. (2016). Hypoxia regulates sumoylation pathways in intervertebral disc cells: implications for hypoxic adaptations. *Osteoarthritis and Cartilage*.

[22] Agrawal A., Gajghate S., Smith H. (2008). Cited2 modulates hypoxia-inducible factor-dependent expression of vascular endothelial growth factor in nucleus pulposus cells of the rat intervertebral disc. *Arthritis and Rheumatism*.

[23] Risbud M. V., Schaer T. P., Shapiro I. M. (2010). Toward an understanding of the role of notochordal cells in the adult intervertebral disc: from discord to accord. *Developmental Dynamics*.

[24] Weiler C., Nerlich A. G., Schaaf R., Bachmeier B. E., Wuertz K., Boos N. (2010). Immunohistochemical identification of notochordal markers in cells in the aging human lumbar intervertebral disc. *European Spine Journal*.

[25] Lee C. R., Sakai D., Nakai T. (2007). A phenotypic comparison of intervertebral disc and articular cartilage cells in the rat. *European Spine Journal*.

[26] Sakai D., Nakai T., Mochida J., Alini M., Grad S. (2009). Differential phenotype of intervertebral disc cells: microarray and immunohistochemical analysis of canine nucleus pulposus and anulus fibrosus. *Spine (Phila Pa 1976)*.

[27] Fujita N., Miyamoto T., Imai J. (2005). CD24 is expressed specifically in the nucleus pulposus of intervertebral discs. *Biochemical and Biophysical Research Communications*.

[28] Rajpurohit R., Risbud M. V., Ducheyne P., Vresilovic E. J., Shapiro I. M. (2002). Phenotypic characteristics of the nucleus pulposus: expression of hypoxia inducing factor-1, glucose transporter-1 and MMP-2. *Cell and Tissue Research*.

[29] Richardson S. M., Ludwinski F. E., Gnanalingham K. K., Atkinson R. A., Freemont A. J., Hoyland J. A. (2017). Notochordal and nucleus pulposus marker expression is maintained by sub-populations of adult human nucleus pulposus cells through aging and degeneration. *Scientific Reports*.

[30] Thorpe A. A., Binch A. L., Creemers L. B., Sammon C., Le Maitre C. L. (2016). Nucleus pulposus phenotypic markers to determine stem cell differentiation: fact or fiction?. *Oncotarget*.

[31] Dai J., Wang H., Liu G., Xu Z., Li F., Fang H. (2014). Dynamic compression and co-culture with nucleus pulposus cells promotes proliferation and differentiation of adipose-derived mesenchymal stem cells. *Journal of Biomechanics*.

[32] Potier E., Ito K. (2014). Can notochordal cells promote bone marrow stromal cell potential for nucleus pulposus enrichment? A simplified *in vitro* system. *Tissue Engineering Part A*.

[33] de Vries S. A., Potier E., van Doeselaar M., Meij B. P., Tryfonidou M. A., Ito K. (2015). Conditioned medium derived from notochordal cell-rich nucleus pulposus tissue stimulates matrix production by canine nucleus pulposus cells and bone marrow-derived stromal cells. *Tissue Engineering. Part A*.

[34] Feng G., Jin X., Hu J. (2011). Effects of hypoxias and scaffold architecture on rabbit mesenchymal stem cell differentiation towards a nucleus pulposus-like phenotype. *Biomaterials*.

[35] Sive J. I., Baird P., Jeziorsk M., Watkins A., Hoyland J. A., Freemont A. J. (2002). Expression of chondrocyte markers by cells of normal and degenerate intervertebral discs. *Molecular Pathology*.

[36] Agrawal A., Guttapalli A., Narayan S., Albert T. J., Shapiro I. M., Risbud M. V. (2007). Normoxic stabilization of HIF-1alpha drives glycolytic metabolism and regulates aggrecan gene expression in nucleus pulposus cells of the rat intervertebral disk. *American Journal of Physiology. Cell Physiology*.

[37] Huang Y. C., Leung V. Y., Lu W. W., Luk K. D. (2013). The effects of microenvironment in mesenchymal stem cell-based regeneration of intervertebral disc. *The Spine Journal*.

[38] Kwon W. K., Moon H. J., Kwon T. H., Park Y. K., Kim J. H. (2017). Influence of rabbit notochordal cells on symptomatic intervertebral disc degeneration: anti-angiogenic capacity on human endothelial cell proliferation under hypoxia. *Osteoarthritis and Cartilage*.

[39] Shi R., Wang F., Hong X. (2015). The presence of stem cells in potential stem cell niches of the intervertebral disc region: an in vitro study on rats. *European Spine Journal*.

[40] Wang F., Cai F., Shi R., Wang X. H., Wu X. T. (2016). Aging and age related stresses: a senescence mechanism of intervertebral disc degeneration. *Osteoarthritis and Cartilage*.

[41] Saito T., Fukai A., Mabuchi A. (2010). Transcriptional regulation of endochondral ossification by HIF-2*α* during skeletal growth and osteoarthritis development. *Nature Medicine*.

[42] Fujita N., Chiba K., Shapiro I. M., Risbud M. V. (2012). HIF-1*α* and HIF-2*α* degradation is differentially regulated in nucleus pulposus cells of the intervertebral disc. *Journal of Bone and Mineral Research*.

[43] Feng C., He J., Zhang Y. (2017). Collagen-derived N-acetylated proline-glycine-proline upregulates the expression of pro-inflammatory cytokines and extracellular matrix proteases in nucleus pulposus cells via the NF-*κ*B and MAPK signaling pathways. *International Journal of Molecular Medicine*.

[44] Sakai D., Andersson G. B. (2015). Stem cell therapy for intervertebral disc regeneration: obstacles and solutions. *Nature Reviews Rheumatology*.

[45] Choi K. S., Harfe B. D. (2011). Hedgehog signaling is required for formation of the notochord sheath and patterning of nuclei pulposi within the intervertebral discs. *Proceedings of the National Academy of Sciences of the United States of America*.

[46] Choi K. S., Lee C., Harfe B. D. (2012). Sonic hedgehog in the notochord is sufficient for patterning of the intervertebral discs. *Mechanisms of Development*.

[47] Risbud M. V., Shapiro I. M. (2011). Notochordal cells in the adult intervertebral disc: new perspective on an old question. *Critical Reviews in Eukaryotic Gene Expression*.

[48] Winkler T., Mahoney E. J., Sinner D., Wylie C. C., Dahia C. L. (2014). Wnt signaling activates Shh signaling in early postnatal intervertebral discs, and re-activates Shh signaling in old discs in the mouse. *PLoS One*.

[49] Minogue B. M., Richardson S. M., Zeef L. A., Freemont A. J., Hoyland J. A. (2010). Transcriptional profiling of bovine intervertebral disc cells: implications for identification of normal and degenerate human intervertebral disc cell phenotypes. *Arthritis Research & Therapy*.

[50] Hotta K., Takahashi H., Satoh N., Gojobori T. (2008). Brachyury-downstream gene sets in a chordate, Ciona intestinalis: integrating notochord specification, morphogenesis and chordate evolution. *Evolution & Development*.

[51] Tang X., Jing L., Richardson W. J. (2016). Identifying molecular phenotype of nucleus pulposus cells in human intervertebral disc with aging and degeneration. *Journal of Orthopaedic Research*.

[52] Tang X., Jing L., Chen J. (2012). Changes in the molecular phenotype of nucleus pulposus cells with intervertebral disc aging. *PLoS One*.

[53] Moll R., Franke W. W., Schiller D. L., Geiger B., Krepler R. (1982). The catalog of human cytokeratins: patterns of expression in normal epithelia, tumors and cultured cells. *Cell*.

[54] Stosiek P., Kasper M., Karsten U. (1988). Expression of cytokeratin and vimentin in nucleus pulposus cells. *Differentiation*.

[55] Sun Z., Wang H. Q., Liu Z. H. (2013). Down-regulated CK8 expression in human intervertebral disc degeneration. *International Journal of Medical Sciences*.

[56] Rutges J., Creemers L. B., Dhert W. (2010). Variations in gene and protein expression in human nucleus pulposus in comparison with annulus fibrosus and cartilage cells: potential associations with aging and degeneration. *Osteoarthritis and Cartilage*.

[57] Rodrigues-Pinto R., Berry A., Piper-Hanley K., Hanley N., Richardson S. M., Hoyland J. A. (2016). Spatiotemporal analysis of putative notochordal cell markers reveals CD24 and keratins 8, 18, and 19 as notochord-specific markers during early human intervertebral disc development. *Journal of Orthopaedic Research*.

